# Focused view CT angiography for selective visualization of stroke related arteries: technical feasibility

**DOI:** 10.1007/s00330-023-09904-6

**Published:** 2023-07-12

**Authors:** Christian Roest, Reina W. Kloet, Maria J. Lamers, Derya Yakar, Thomas C. Kwee

**Affiliations:** 1 Medical Imaging Center, Department of Radiology, University of Groningen, University Medical Center Groningen, Groningen, the Netherlands; 2 Department of Radiology, Netherlands Cancer Institute, Amsterdam, the Netherlands

**Keywords:** Computed tomography angiography, Diagnosis, Deep learning, Ischemic stroke

## Abstract

**Objectives:**

This study investigated the technical feasibility of focused view CTA for the selective visualization of stroke related arteries.

**Methods:**

A total of 141 CTA examinations for acute ischemic stroke evaluation were divided into a set of 100 cases to train a deep learning algorithm (dubbed “focused view CTA”) that selectively extracts brain (including intracranial arteries) and extracranial arteries, and a test set of 41 cases. The visibility of anatomic structures at focused view and unmodified CTA was assessed using the following scoring system: 5 = completely visible, diagnostically sufficient; 4 = nearly completely visible, diagnostically sufficient; 3 = incompletely visible, barely diagnostically sufficient; 2 = hardly visible, diagnostically insufficient; 1 = not visible, diagnostically insufficient.

**Results:**

At focused view CTA, median scores for the aortic arch, subclavian arteries, common carotid arteries, C1, C6, and C7 segments of the internal carotid arteries, V4 segment of the vertebral arteries, basilar artery, cerebellum including cerebellar arteries, cerebrum including cerebral arteries, and dural venous sinuses, were all 4. Median scores for the C2 to C5 segments of the internal carotid arteries, and V1 to V3 segments of the vertebral arteries ranged between 3 and 2. At unmodified CTA, median score for all above-mentioned anatomic structures was 5, which was significantly higher (*p* < 0.0001) than that at focused view CTA.

**Conclusion:**

Focused view CTA shows promise for the selective visualization of stroke-related arteries. Further improvements should focus on more accurately visualizing the smaller and tortuous internal carotid and vertebral artery segments close to bone.

**Clinical relevance:**

Focused view CTA may speed up image interpretation time for LVO detection and may potentially be used as a tool to study the clinical relevance of incidental findings in future prospective long-term follow-up studies.

**Key Points:**

• *A deep learning–based algorithm (“focused view CTA”) was developed to selectively visualize relevant structures for acute ischemic stroke evaluation at CTA.*

• *The elimination of unrequested anatomic background information was complete in all cases.*

• *Focused view CTA may be used to study the clinical relevance of incidental findings.*

**Supplementary Information:**

The online version contains supplementary material available at 10.1007/s00330-023-09904-6.

## Introduction

Acute ischemic stroke, a major cause of both disability and death, has an incidence of approximately 94.5 per 100,000 people and affected an estimated 7.63 million people worldwide in 2019 [1].

Computed tomography (CT) plays an important role for treatment selection in acute ischemic stroke [2]. CT angiography (CTA) is used to identify large vessel occlusions (LVOs) suitable for endovascular thrombectomy (EVT), to assess the cervical vessels for the presence of dissection, critical stenoses, or other vascular variants or abnormalities that may complicate EVT, and can be used as a roadmap for the EVT [2]. The CTA examination should have an anatomic coverage from the origins of the cervical vessels at the aortic arch extending to the cranial vertex [2].

Although the sole purpose of the CTA examination is to visualize the intracranial and extracranial arteries, many other organs are also visualized due to its anatomic coverage. Visualization of structures outside the brain and major arteries at CTA may increase interpretation time of the radiologist. Another issue is that incidental findings (i.e., unanticipated findings not related to the original diagnostic inquiry) at CTA in ischemic stroke patients are common, with one recent study reporting 15.4% of acute ischemic patients to have an incidental finding of major clinical relevance [3]. They increase healthcare costs, while the true clinical relevance of the majority of these incidentalomas, and whether or not they should require any additional investigations and/or be treated at all, remains unclear [4].

Deep learning has revolutionized image post-processing and is used for many medical image segmentation tasks [5]. For example, a previous study reported that fully automated segmentation of the cerebral arteries is feasible [6]. We hypothesize that deep learning–based image segmentation can be used to selectively extract brain (including intracranial arteries) and extracranial arteries from a CTA examination that are relevant for ischemic stroke evaluation, while keeping all other unrequested anatomic structures hidden. This approach, which we dub “focused view CTA,” may speed up image interpretation in acute ischemic stroke patients and provides a means to investigate the relevance of incidental findings at CTA.

The purpose of this study was to investigate the technical feasibility of focused view CTA for the selective visualization of stroke related arteries.

## Materials and methods

### Study design and patients

This retrospective study was approved by the local institutional review board and the requirement for informed consent was waived. A total of 150 consecutive patients who underwent CT for acute ischemic stroke evaluation between 25 September and 24 December 2021 were potentially eligible for inclusion in this study. Of these 150 patients, 8 were excluded because CTA was not performed, and 1 was excluded because of diagnostically relevant motion artefacts. The remaining 141 patients were randomly divided into a training set of 100 cases and a separately held test set of 41 cases.

### CTA acquisition

CTA was performed using a multi-detector row CT system (SOMATOM Definition Edge, Siemens Healthineers). After intravenous administration of 50 mL of iomeprol (Iomeron 350, Bracco Imaging) at a flow rate of 6 mL/s (same contrast agent dosage and injection rate for all adult patients in this study), and a scan delay of 2 s after bolus triggering (threshold of 100 Hounsfield units (HUs) in the proximal descending thoracic aorta), CTA images were acquired from the aortic arch to the cranial vertex, using the following settings: tube voltage of 100 kV, gantry rotation time of 0.285 s, collimation of 0.6 mm, pitch factor of 0.8, and automated exposure control switched on during all acquisitions (CARE Dose 4D; Siemens). CTA images were iteratively reconstructed (ADMIRE, Siemens Healthineers) with a slice thickness/increment of 0.75/0.5 mm. Estimated effective dose was approximately 0.6 mSv. Unenhanced CT and CT perfusion (CTP) images of the brain were also acquired, but these were not evaluated in this study.

### Training set for focused view CTA

**Fig. 1:**
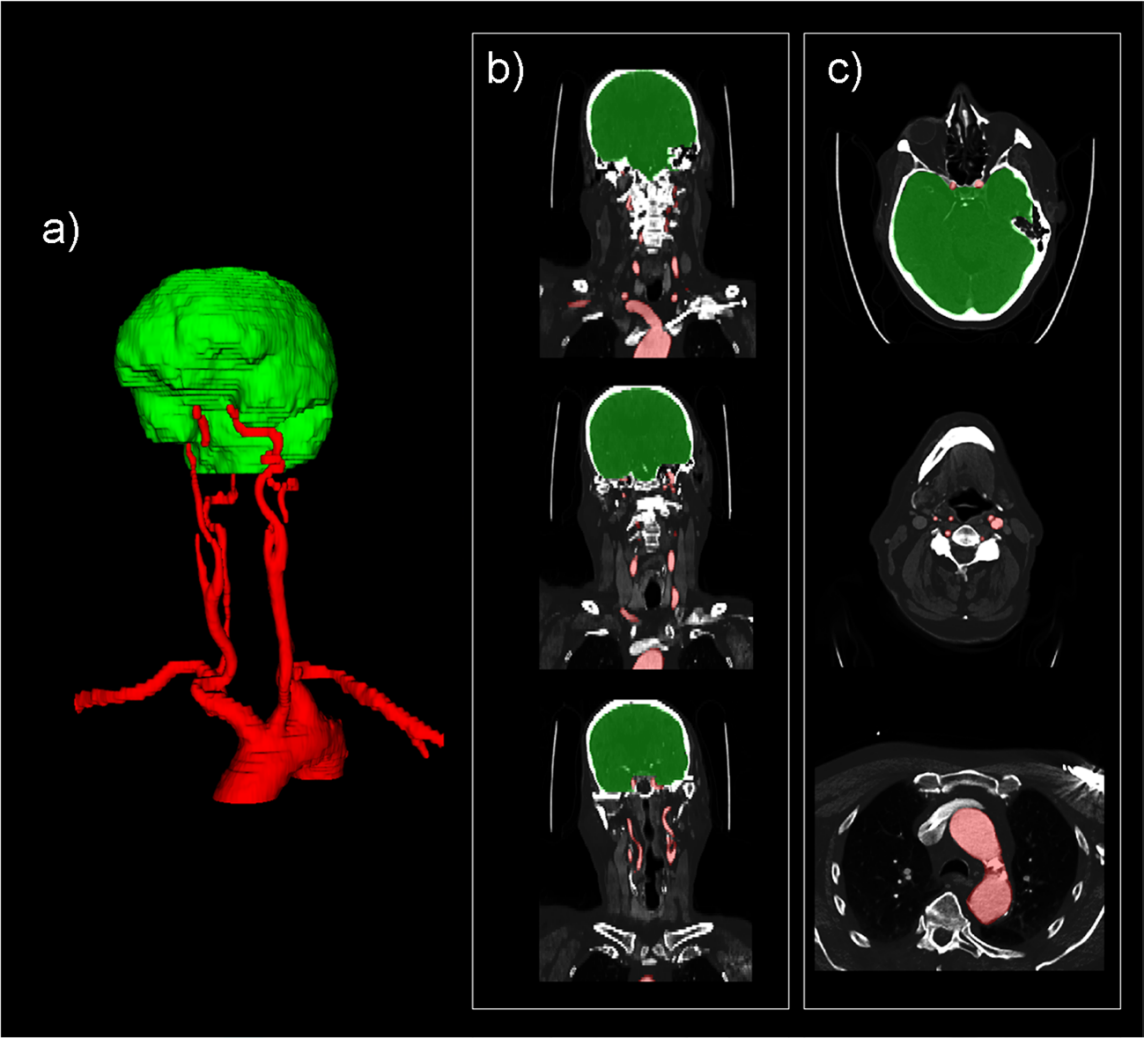
Example of a segmentation in the training set to selectively extract brain (including intracranial arteries) and extracranial arteries for focused view CTA. Step 1 concerned the segmentation of the cranial cavity and the proximal spinal canal until the level of the transition from the V4 to V3 segment of the vertebral arteries (green segmentation); step 2 concerned the segmentation of the aortic arch, subclavian arteries, common carotid arteries, proximal external carotid arteries, internal carotid arteries until the level of the transition from the cavernous to the cerebral segment, and vertebral arteries until the transition from the V3 to V4 segment (red segmentation). Segmentations in three-dimensional view (**a**), on some coronal slices (**b**), and on some axial slices (**c**) are shown

The CTA examinations of the 100 training cases were segmented by a board-certified radiologist (TCK) using dedicated software (ITK-SNAP, version 3.8.0 [7]). Segmentation was performed in two steps: step 1 concerned the segmentation of the cranial cavity and the proximal spinal canal until the level of the transition from the V4 to V3 segment of the vertebral arteries; step 2 concerned the segmentation of the aortic arch, subclavian arteries, common carotid arteries, proximal external carotid arteries, internal carotid arteries until the level of the transition from the cavernous to the cerebral segment, and vertebral arteries until the transition from the V3 to V4 segment (Fig. 1).

### Deep learning

A deep neural network (DNN) was developed to segment the previously described anatomic structures at CTA. A patch-based approach was adopted to enable full-scale segmentation of imaging volumes with varying numbers of slices. Cross-validation (CV) was performed on the training set to train five separate deep learning segmentation models. Randomly sampled scan crops from the training fold (90%, 90 scans) were used as training samples, while samples from the validation fold (10%, 10 scans) were used to monitor the performance between epochs. The mean categorical dice loss over the two foreground classes was used as the loss function [8]. To prevent overfitting, training was stopped after 50 epochs without improvement in validation performance. Predictions were generated using a sliding-window, and overlapping predictions were averaged for each voxel. The best models for each fold were combined in an averaging ensemble to improve the consistency of the generated segmentations. The dice coefficient was calculated for each validation fold to assess the quality of the generated segmentations. The deep learning model was implemented using Python 3.7.4 and TensorFlow 2.2.0. An overview of the deep learning pipeline is shown in Supplementary Fig. 1.

### Hyperparameter optimization

Extensive hyperparameter optimization was performed to determine the optimal configuration for our deep learning pipeline. Tuned parameters included the DNN architecture, resampling, preprocessing, and data augmentation strategies. Seventy-five optimization trials, consisting of a single CV fold and a maximum of 50 epochs, were completed for different hyperparameter configurations. The configuration that achieved the best mean dice score on the validation set was used to train the final models as described above. An overview of the parameter search space and optimal values for each hyperparameter is shown in Table 1. Hyperparameter optimization was performed using Optuna 2.10.0.

**Table 1:** Overview of the parameter search space and optimal values for each hyperparameter

Parameter	Search space	Optimal value
Preprocessing		
In plane voxel spacing	0.5/0.7/1.0	0.7
Normalization strategy	Z-norm/divide by 1000	Z-norm
Deep learning		
Window shape (X, Y)	160–256 (step size: 32)	160
Window shape (Z)	16–64 (step size: 16)	48
U-Net architecture	Basic U-Net/Dual Attn. U-Net	Dual attention
Batch size	1–15 (step size: 1)	13
Learning rate	1e^−4^–1e^−3^	4e^−4^
Kernel regularization (L2)	1e^−4^–1e^−3^	2e^−4^
Optimizer	Adam/RMSProp	RMSProp
Instance normalization	Enabled/disabled	Enabled
Data augmentation		
Rotation frequency	0.0–0.5	0.25
Tilt frequency	0.0–0.5	0.04
Noise frequency	0.0–0.7	0.60
Noise factor	1e^−4^–1e^−2^	0.003

### CTA modification

The deep learning ensemble was applied to each image in the unannotated test data, to generate segmentations of the brain (including intracranial arteries) and extracranial arteries. Focused view CTA scans were created by setting the intensities of voxels outside of the generated segmentation equal to − 1000. Focused view and unmodified CTA scans were subsequently exported to DICOM format.

### CTA evaluation

The focused view and unmodified CTA examinations of the 41 test cases were evaluated by a board-certified neuroradiologist (R.W.K.), who performed all evaluations without any clinical information and without unenhanced CT and CTP data. Focused view CTA and unmodified CTA evaluations were evaluated separately in different reading sessions. For the purpose of inter-reader agreement analysis, a second neuroradiologist (M.J.L.) also evaluated all focused view CTA examinations, in an independent and blinded manner. The visibility of the extracranial and intracranial vessels at both focused view and unmodified CTA was assessed using a 5-point scale: score 5 = completely visible, diagnostically sufficient; score 4 = nearly completely visible, diagnostically sufficient; score 3 = incompletely visible, barely diagnostically sufficient; score 2 = hardly visible, diagnostically insufficient; score 1 = not visible, diagnostically insufficient. This scoring system was applied to the following anatomic structures: aortic arch at the level of the origin of the cervical vessels, right and left subclavian artery, right and left common carotid artery, C1, C2, C3, C4, C5, C6, and C7 segments of the right and left internal carotid artery [9], V1, V2, V3, and V4 segments of the right and left vertebral artery [10], basilar artery, cerebellum including cerebellar arteries, cerebrum including cerebral arteries, and dural venous sinuses. In addition, the elimination of unrequested anatomic background information (i.e., all other anatomic structures than aortic arch, subclavian arteries, carotid arteries, vertebral arteries, basilar artery, cerebellum, and cerebrum) was assessed at focused view CTA. Note that although the benefit of EVT for posterior circulation stroke is currently uncertain [11], for completeness we also included the cerebrum/cerebellum including cerebral arteries in our evaluation. For this evaluation we focused on both the cerebellar parenchyma and the cerebellar arteries as a whole. An overview of the scoring system is shown in Supplementary Fig. 2.

### Data analysis

The visibility scores of the various anatomic structures (aortic arch at the level of the origin of the cervical vessels, subclavian arteries, common carotid arteries, C1 to C7 segments of the internal carotid arteries, V1 to V4 segments of the vertebral arteries, basilar artery, cerebellum including cerebellar arteries, cerebrum including cerebral arteries, and dural venous sinuses) were compared between focused view CTA and unmodified CTA, using Wilcoxon tests. *p*-values < 0.05 were considered statistically significant. Inter-reader agreement was assessed using Cohen’s weighted kappa. All statistical analyses were executed using MedCalc version 19.1.6 software (MedCalc).

## Results

### Patients

The 100 training cases consisted of 61 men and 39 women, with a mean age of 69.2 ± 12.2 years (range: 40–92 years). The 41 test cases consisted of 29 men and 12 women, with a mean age of 64.7 ± 13.1 years (range: 31–86 years). An overview of the patient characteristics for both datasets, including the presence of LVO and atherosclerotic plaques, is presented in Table 2. No significant differences in patient characteristics were found between training and test datasets.

**Table 2:** Characteristics of patients in the training set and test set. All patients presented with a clinical suspicion of acute ischemic stroke, for which CTA was performed

Variable	Training set (*n* = 100)	Test set (*n* = 41)	*p*-value
Age (years)	72.4 (IQR: 59.6–78.6)	65.2 (IQR: 56.0–75.9)	0.087^a^
Gender (male/female)	61/39	29/12	0.369^b^
LVO (yes/no)	24^c^/76	6^d^/35	0.314^b^
Any atherosclerotic plaque with > 50% stenosis in any part of the left internal carotid artery (yes/no)^e^	6/86	3/38	1^b^
Any atherosclerotic plaque with > 50% stenosis in any part of the right internal carotid artery (yes/no)^e^	10/89	3/38	0.844^b^
Any atherosclerotic plaque with > 50% stenosis in any of part of the left vertebral artery (yes/no)^e^	3/97	3/37	0.468^b^
Any atherosclerotic plaque with > 50% stenosis in any of part of the right vertebral artery (yes/no)^e^	6/94	2/39	1^b^

^a^Mann-Whitney test

^b^Chi-square test

^c^Locations of LVOs at CTA: M2 and M3 segments of the middle cerebral artery (*n* = 4), M3 segment of the middle cerebral artery (*n* = 4), M1 segment of the middle cerebral artery (*n* = 2), extracranial and intracranial internal carotid artery (*n* = 2), extracranial internal carotid artery, intracranial internal carotid artery, and M1 segment of the middle cerebral artery (*n* = 1), extracranial internal carotid artery, intracranial internal carotid artery, and M1 and M2 segments of the middle cerebral artery (*n* = 1), extracranial internal carotid artery, intracranial internal carotid artery, and M1, M2, and M3 segments of the middle cerebral artery (*n* = 1), extracranial internal carotid artery, intracranial internal carotid artery, and M3 segment of the middle cerebral artery (*n* = 1), intracranial internal carotid artery and M1 segment of the middle cerebral artery (*n* = 1), intracranial internal carotid artery, M1 segment of the middle cerebral artery, and A1 segment of the anterior cerebral artery (*n* = 1), intracranial internal carotid artery, and M1, M2, and M3 segments of the middle cerebral artery (*n* = 1), M1 and M2 segments of the middle cerebral artery (*n* = 1), M1 segment of the middle cerebral artery and A1 segment of the anterior cerebral artery (*n* = 1), M2 segment of the middle cerebral artery (*n* = 1), basilar artery (*n* = 1), and P1 segment of the posterior cerebral artery (*n* = 1)

^d^Locations of LVOs at CTA: M2 segment of the middle cerebral artery (*n* = 2), M2 and M3 segments of the middle cerebral artery (*n* = 1), A1 segment of the anterior cerebral artery (*n* = 1), intracranial vertebral artery (*n* = 1), and P1 and P2 segments of the posterior cerebral artery (*n* = 1)

^e^Arteries with a complete occlusion (as described in notes ^c^ and ^d^) were excluded from this analysis

*CTA*, computed tomography angiography; *IQR*, interquartile range; *LVO*, large vessel occlusion

### Segmentation time and quality

The average time required for the conversion of unmodified CTA to focused view CTA was 2:03 min (SD = 1:03 min). The median Dice score was 0.99 (interquartile range [IQR] = 0.99–1) for the brain and intracranial arteries, and 0.94 (IQR = 0.92–0.95) for the extracranial arteries.

### Visibility of anatomic structures at focused view CTA

**Fig. 2:**
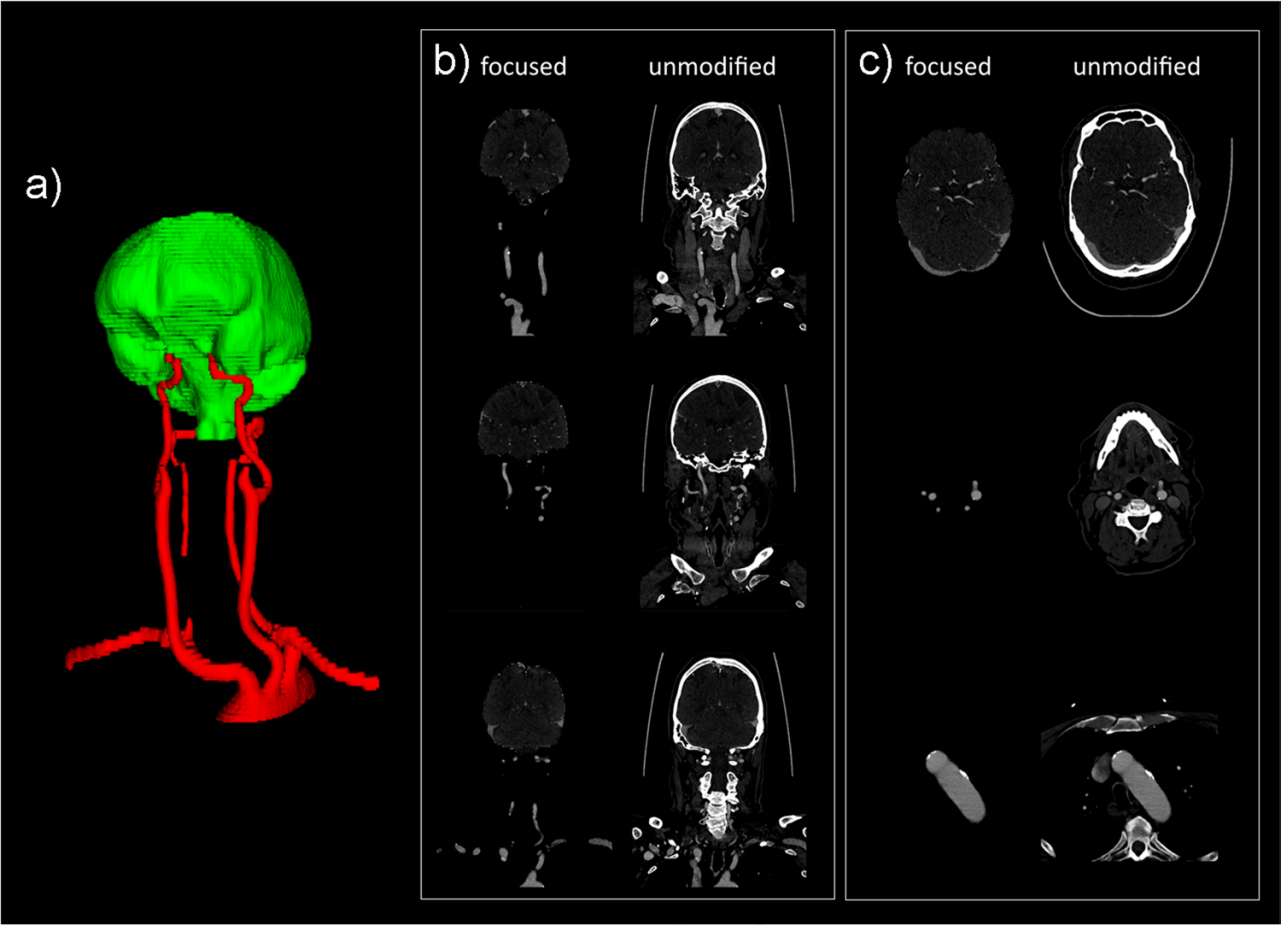
First example of focused view CTA in the test set. Three-dimensional segmentation (**a**), coronal focused view CTA slices next to unmodified CTA slices (**b**), and axial focused view CTA slices next to unmodified CTA slices (**c**) are shown. The full focused view CTA dataset is shown in supplementary (video) file 1

**Fig. 3:**
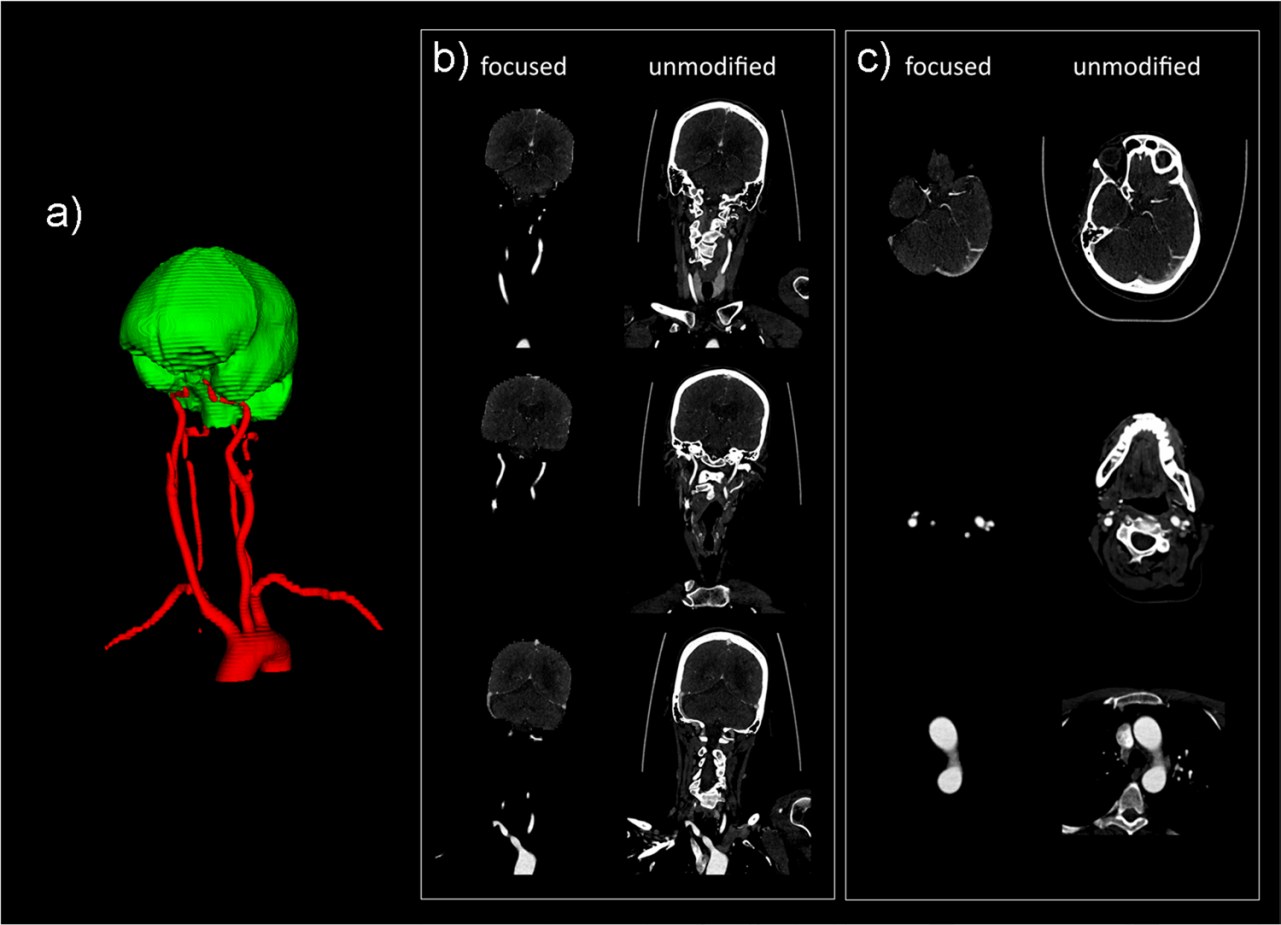
Second example of focused view CTA in the test set. Three-dimensional segmentation (**a**), coronal focused view CTA slices next to unmodified CTA slices (**b**), and axial focused view CTA slices next to unmodified CTA slices (**c**) are shown. The full focused view CTA dataset is shown in supplementary (video) file 2

**Fig. 4:**
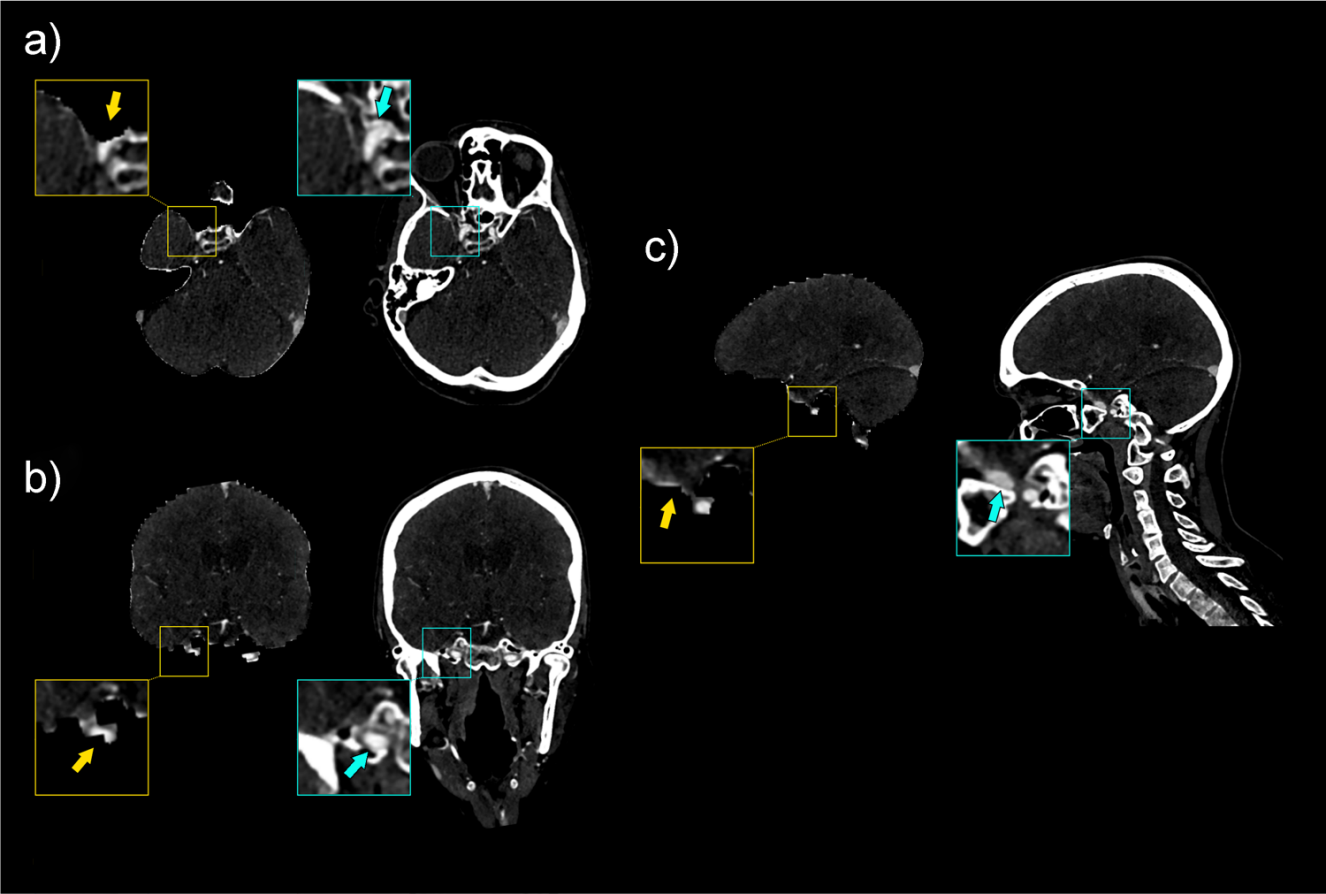
Example of incomplete visualization of the C5 segment of a right internal carotid artery in a test set case, shown in axial (**a**), coronal (**b**), and sagittal (**c**) directions, along with zoomed images, and indicated with arrows

**Fig. 5:**
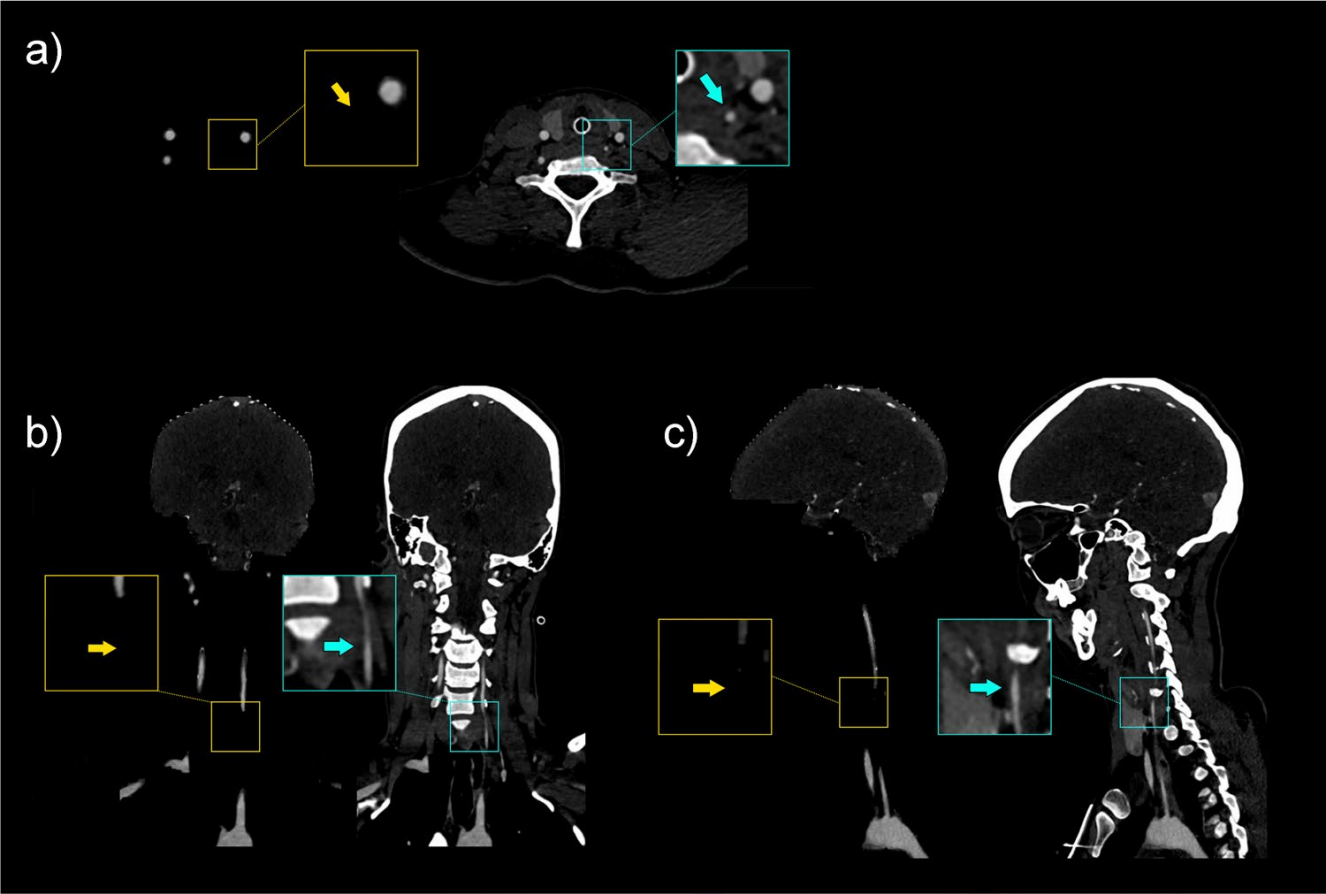
Example of incomplete visualization of the V1 segment of a left (hypoplastic) vertebral artery that has a slightly ventral course in a test set case, shown in axial (**a**), coronal (**b**), and sagittal (**c**) directions, along with zoomed images, and indicated with arrows

Median scores for the aortic arch, subclavian arteries, common carotid arteries, C1, C6, and C7 segments of the internal carotid arteries, V4 segment of the vertebral arteries, basilar artery, cerebellum including cerebellar arteries, cerebrum including cerebral arteries, and dural venous sinuses, were all 4. Median scores for the C2 to C5 segments of the internal carotid arteries, and V1 to V3 segments of the vertebral arteries ranged between 3 and 2 (Table 3). The elimination of unrequested anatomic background information was complete in all cases. Inter-reader agreement was moderate (κ = 0.5). Representative examples are shown in Figs. 2 and 3, with corresponding Supplementary video files 1 and 2. In addition, two (close-up) examples of focal incomplete vessel visualizations are shown in Figs. 4 and 5.

**Table 3:** Comparison of visibility of various anatomic structure(s) between focused view CTA and unmodified CTA

Anatomic structure(s)	Focused view CTA^a^	Unmodified CTA^a^	*p*-value^b^
Aortic arch at the level of the origin of the cervical vessels	4 (IQR: 4–4, range: 2–4)	5 (IQR: 5–5, range: 4–5)	< 0.0001
Right subclavian artery	4 (IQR: 3–4, range: 1–4)	5 (IQR: 4.5–5, range: 4–5)	< 0.0001
Left subclavian artery	4 (IQR: 3–4, range: 1–4)	5 (IQR: 4–5, range: 3–5)	< 0.0001
Right common carotid artery	4 (IQR: 4–4, range: 1–4)	5 (IQR: 5–5, range: 4–5)	< 0.0001
Left common carotid artery	4 (IQR: 4–4, range: 1–4)	5 (IQR: 5–5, range: 4–5)	< 0.0001
Right internal carotid artery			
-C1	4 (IQR: 4–4, range: 1–4)	5 (IQR: 5–5, range: 4–5)	< 0.0001
-C2	3 (IQR: 3–3, range: 1–4)	5 (IQR: 5–5, range: 3–5)	< 0.0001
-C3	3 (IQR: 3–3, range: 2–4)	5 (IQR: 5–5, range: 3–5)	< 0.0001
-C4	3 (IQR: 2.5–3, range: 1–4)	5 (IQR: 5–5, range: 5–5)	< 0.0001
-C5	3 (IQR: 2–3, range: 1–4)	5 (IQR: 5–5, range: 4–5)	< 0.0001
-C6	4 (IQR: 4–4, range: 1–4)	5 (IQR: 5–5, range: 5–5)	< 0.0001
-C7	4 (IQR: 4–4, range: 4–4)	5 (IQR: 5–5, range: 5–5)	< 0.0001
Left internal carotid artery			
-C1	4 (IQR: 4–4, range: 1–4)	5 (IQR: 5–5, range: 5–5)	< 0.0001
-C2	3 (IQR: 3–3, range: 2–4)	5 (IQR: 5–5, range: 3–5)	< 0.0001
-C3	3 (IQR: 3–3.5, range: 2–4)	5 (IQR: 5–5, range: 3–5)	< 0.0001
-C4	3 (IQR: 2–3, range: 1–4)	5 (IQR: 5–5, range: 5–5)	< 0.0001
-C5	2.5 (IQR: 2–3, range: 1–4)	5 (IQR: 5–5, range: 5–5)	< 0.0001
-C6	4 (IQR: 4–4, range: 1–4)	5 (IQR: 5–5, range: 5–5)	< 0.0001
-C7	4 (IQR: 4–4, range: 4–4)	5 (IQR: 5–5, range: 5–5)	< 0.0001
Right vertebral artery			
V1	3 (IQR: 1–4, range: 1–4)	5 (IQR: 4.5–5, range: 2–5)	< 0.0001
V2	3 (IQR: 3–4, range: 1–4)	5 (IQR: 5–5, range: 3–5)	< 0.0001
V3	3 (IQR: 1–3, range: 1–4)	5 (IQR: 5–5, range: 4–5)	< 0.0001
V4	4 (IQR: 4–4, range: 2–4)	5 (IQR: 5–5, range: 1–5)	< 0.0001
Left vertebral artery			
V1	3 (IQR: 1–4, range: 1–4)	5 (IQR: 4.5–5, range: 1–5)	< 0.0001
V2	3 (IQR: 2–4, range: 1–4)	5 (IQR: 5–5, range: 1–5)	< 0.0001
V3	2 (IQR: 1–3, range: 1–4)	5 (IQR: 5–5, range: 1–5)	< 0.0001
V4	4 (IQR: 4–4, range: 1–4)	5 (IQR: 5–5, range: 1–5)	< 0.0001
Basilar artery	4 (IQR: 4–4, range: 1–4)	5 (IQR: 5–5, range: 4–5)	< 0.0001
Cerebellum including cerebellar arteries	4 (IQR: 3.5–4, range: 2–4)	5 (IQR: 5–5, range: 3–5)	< 0.0001
Cerebrum including cerebral arteries	4 (IQR: 4–4, range: 4–4)	5 (IQR: 5–5, range: 5–5)	< 0.0001
Dural venous sinuses	4 (IQR: 4–4, range: 4–4)	5 (IQR: 5–5, range: 5–5)	< 0.0001

^a^Median value with interquartile range

^b^Wilcoxon test

Score 5 = completely visible (diagnostically sufficient)

Score 4 = nearly completely visible (diagnostically sufficient)

Score 3 = incompletely visible (barely diagnostically sufficient)

Score 2 = hardly visible (diagnostically insufficient)

Score 1 = not visible (diagnostically insufficient)

*IQR*, interquartile range

### Comparison with unmodified CTA

At unmodified CTA, median score for all anatomic structures was 5, which was significantly higher (*p* < 0.0001) than that at focused view CTA (Table 3).

## Discussion

The results of this study show that the developed deep learning algorithm is able to segment and selectively display the structures of interest from the aortic arch to cranial vertex that matter for the CTA evaluation of patients with acute ischemic stroke, while eliminating unrequested anatomic background information. Overall, the aortic arch, the subclavian arteries, the proximal and distal internal carotid arteries (C1, C6, and C7 segments), the distal vertebral artery (V4 segment), basilar artery, and intracranial structures (including arteries and dural venous sinuses) were satisfactorily visualized at focused field CTA. However, the segmentations of the middle part of the internal carotid arteries (C2 to C5 segments) and the proximal and middle parts of the vertebral arteries (V1 to V3 segments) were generally rated as barely diagnostically sufficient to diagnostically insufficient. This is probably related to the relatively small caliber of these arteries, combined with their often tortuous course and their close vicinity to bone (vertebrae and skull base, with HUs approaching those of opacified arteries) in these locations. Future technical efforts should be spent on improving the visualization of the C2 to C5 segments of the internal carotid arteries and V1 to V3 segments of the vertebral arteries. This may not only be realized by training the algorithm with more cases, but perhaps even more by sophisticating the algorithm with methods such as automatic bone removal and vessel tracking [12–15]. Nevertheless, this limitation of the current algorithm may be considered minor, because the far majority of treatable LVOs are located elsewhere, i.e., typically M1 and M2 segments of the middle cerebral artery, at the T-junction where the internal carotid artery bifurcates into the middle cerebral artery, at the C1 segment of the internal carotid artery (although this may proceed more distally), and in the basilar artery, which were all well visualized at focused view CTA. A potential exception concerns isolated dissection of the petrous (C2) segment of the internal carotid artery [16], which may be missed with the current focused view CTA algorithm.

The conversion from unmodified CTA to focused view CTA only took 2 min on average, but with several technical optimizations (such as batching and quantization) the conversion time may be further reduced to minimize any diagnostic delay. Moreover, the availability of a larger training dataset may reduce the need for ensembling of the deep learning predictions and could decrease the conversion time by up to five times. All code used for training and generating segmentations (which required > 200 h of segmentation time by a radiologist) has been made available at the following repository: https://github.com/0xC4/focused-view (note that this will be available after publication of this article). This allows researchers to further develop the focused view CTA approach.

Focused view CTA may have several applications, once optimized for clinical use. First, it may speed up image interpretation time for LVO detection. Second, the unrequested anatomic background information that has been eliminated on the focused view CTA scan may be handled in several ways. In busy practices that are relatively understaffed during on-call hours, the full, unmodified CTA examination may perhaps be reviewed for extracranial and extravascular findings at a later moment when time is less pressing. On another note, focused view CTA may potentially be used to investigate the clinical relevance of incidental findings. This may perhaps be achieved by future prospective long-term follow-up studies in which acute ischemic stroke patients are randomized to either undergo unmodified CTA or to undergo focused view CTA. Unmodified CTA may detect incidental findings in the field of view that may have to be acted upon, whereas focused view CTA may allow many incidental findings in the native field of view to be deliberately hidden from anyone and their natural clinical course to be followed. Whether or not it would be feasible to perform such a study (e.g., in terms of ethical review board approval, patient participation and consent, and sufficient follow-up time) requires further research. Since the advent of spiral and multi-detector row CT more than 25 years ago, CTA has gradually evolved into an accepted minimally invasive and less costly alternative to catheter angiography [17]. Previous studies have demonstrated the feasibility of removing bone from CTA data to improve the assessment of arteries close to bone [12, 13]. Other techniques, including deep learning models, have been reported to selectively visualize the intracranial arteries [6, 18–20]. However, these techniques exclude brain parenchyma and do not include the extracranial arteries until the aortic arch. Nevertheless, it should be noted that for the sole purpose of detecting a thrombus in the intracranial arteries, it would not be necessary to visualize the brain parenchyma on CTA. However, unenhanced CT and CT perfusion are generally also part of the CT stroke protocol, and the same mask that is used for CTA may perhaps also be applied to the unenhanced CT and CT perfusion scans (on which the visualization of brain parenchyma is paramount), which may potentially be easier from a workflow perspective. Other than bone removal and intracranial artery segmentation, no other methods have been reported on how to selectively visualize only those anatomic structures between the aortic arch and cranial vertex at CTA that are relevant to acute ischemic stroke evaluation.

The present study had some limitations. First, the neuroradiologists who evaluated the test cases had never seen any focused view CTA images before. The neuroradiologist who evaluated both focused view and unmodified CTA scans realized that she generally assigned lower scores to the visibility of the relevant anatomic structures at focused field CTA than was actually the case after having reviewed the entire dataset of both focused view and unmodified CTA scans. The lack of experience with focused view CTA may also be a partial explanation for the moderate inter-reader agreement. Second, this study focused on technical performance. The diagnostic performance of focused view CTA for LVO detection, its effect on workflow processes and speed, and its influence on patient outcome were not assessed. The same applies to the effects of omitting unrequested anatomic background information and incidental imaging findings on patient management and outcome. Third, the dataset used for evaluation was relatively small, and consisted of patients scanned on a single scanner in a single center, which may limit the generalizability of our model.

In conclusion, focused view CTA shows promise for the selective visualization of stroke related arteries, which may eventually be used for acute ischemic stroke evaluation. Further technical improvements should particularly focus on more accurately visualizing the smaller and tortuous internal carotid and vertebral artery segments close to bone.

### Supplementary Information

Below is the link to the electronic supplementary material.

Supplementary file1 (PDF 395 kb)

Supplementary file2 (MP4 4657 kb)

Supplementary file3 (MP4 4957 kb)

## Abbreviations

CT: Computed tomography
CTA: Computed tomography angiography
CTP: Computed tomography perfusion
CV: Cross-validation
DNN: Deep neural network
EVT: Endovascular thrombectomy
HU: Hounsfield unit
IQR: Interquartile range
LVO: Large vessel occlusion

## Declarations

### Guarantor

The scientific guarantor of this publication is C. Roest.

### Statistics and biometry

No complex statistical methods were necessary for this paper.

### Study subjects or cohorts overlap

NA

### Methodology

• Retrospective

• Experimental

• performed at one institution
