# Supplementary material for: Focused view CT angiography for selective visualization of stroke related arteries: technical feasibility
Source: Eur Radiol. 2023 Jul 12;33(12):9099–108. doi: 10.1007/s00330-023-09904-6 (PMC10667412; doi:10.1007/s00330-023-09904-6)
Supplement: Supplementary file 1 — Supplementary file1 (PDF 395 kb) [file 330_2023_9904_MOESM1_ESM.pdf]

Focused view CT angiography for selective visualization of stroke related arteries: technical feasibility

Electronic Supplementary Material

Supplementary table

Supplementary table 1 Mean dice scores and standard deviations calculated for the validation partition of each cross-validation fold.

fold number	external arteries	brain
1	0.91 (0.06)	0.992 (0.003)
2	0.93 (0.02)	0.993 (0.0008)
3	0.93 (0.03)	0.994 (0.001)
4	0.93 (0.04)	0.994 (0.001)
5	0.94 (0.02)	0.993 (0.002)
Overall	0.93 (0.03)	0.993 (0.002)

Supplementary figures

Supplementary figure 1 An overview of the deep learning pipeline: a) patch-based training of the 3D deep learning segmentation model using cross-validation; b) conversion from unmodified to focused view CTA scan using an ensemble of trained segmentation model.

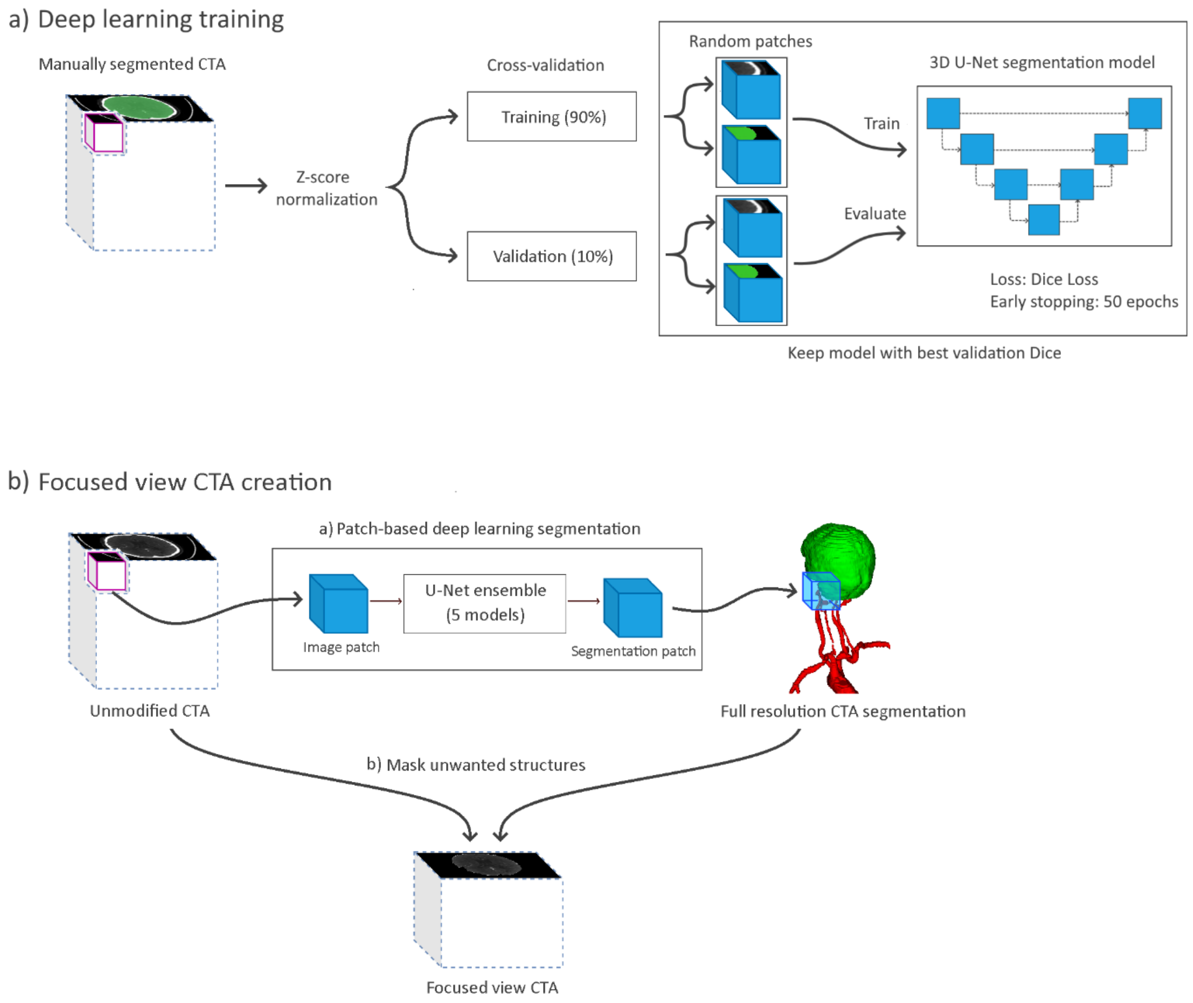

Supplementary figure 2 An overview of the scoring system used by the two neuroradiologists to evaluate the quality of the focused view CTA scans.

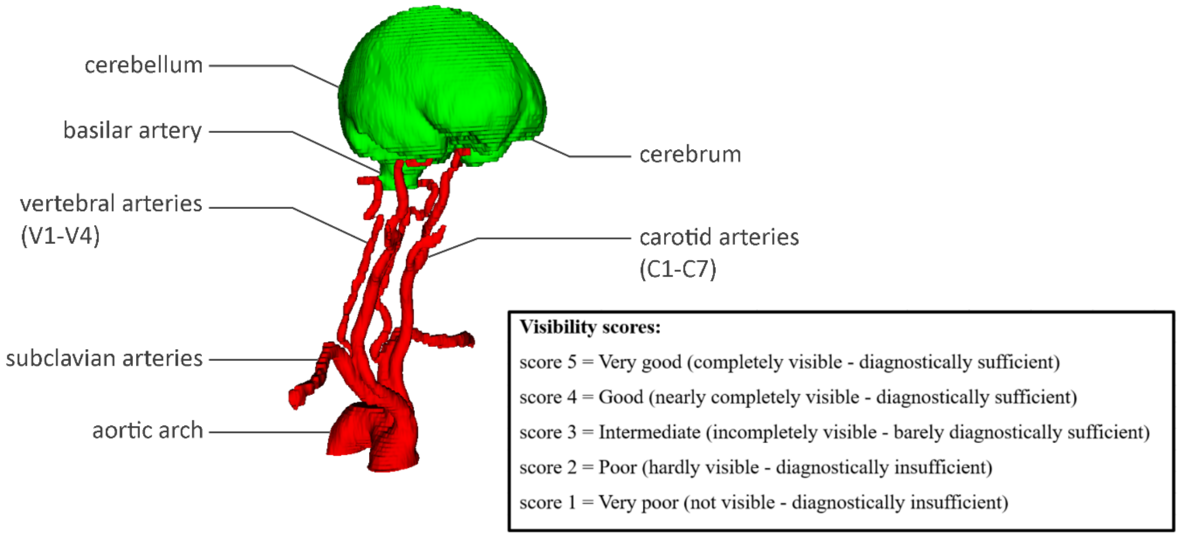

Supplementary videos

Supplementary (video) file 1 All coronal and axial focused view CTA slices (left) shown next to next to unmodified CTA slices (right) of example 1.

Supplementary (video) file 2 All coronal and axial focused view CTA slices (left) next to unmodified CTA slices (right) of example 2.
